# Basic Characteristics of Ionic Liquid-Gated Graphene FET Sensors for Nitrogen Cycle Monitoring in Agricultural Soil

**DOI:** 10.3390/bios15010055

**Published:** 2025-01-16

**Authors:** Naoki Shiraishi, Jian Lu, Fatin Bazilah Fauzi, Ryo Imaizumi, Toyohiro Tsukahara, Satoshi Mogari, Shosuke Iida, Yusuke Matsukura, Satoshi Teramoto, Keisuke Yokoi, Izumi Ichinose, Mutsumi Kimura

**Affiliations:** 1 National Agriculture and Food Research Organization (NARO), 3-1-1 Kannondai, Tsukuba 305-8517, Ibaraki, Japan; futembajiraf647@affrc.go.jp; 2 National Institute of Advanced Industrial Science and Technology (AIST), 1-2-1 Namiki, Tsukuba 305-8564, Ibaraki, Japan; jian-lu@aist.go.jp; 3 Department of Chemistry and Materials, Faculty of Textile Science and Technology, Shinshu University, 3-15-1 Tokida, Ueda 386-8567, Nagano, Japan; 23fs403f@shinshu-u.ac.jp (R.I.); mkimura@shinshu-u.ac.jp (M.K.); 4 Niterra Co., Ltd., 2808 Iwasaki, Komaki 485-8510, Aichi, Japan; toyohiro.tsukahara@niterragroup.com (T.T.); satoshi.mogari@niterragroup.com (S.M.); shosuke.iida@niterragroup.com (S.I.); yusuke.matsukura@niterragroup.com (Y.M.); satoshi.teramoto@niterragroup.com (S.T.); keisuke.yokoi@niterragroup.com (K.Y.); 5 National Institute for Materials Science (NIMS), 1-1 Namiki, Tsukuba 305-0044, Ibaraki, Japan; ichinose.izumi@nims.go.jp

**Keywords:** ionic liquid, graphene FET, nitrogen cycle monitoring, N_2_O, agricultural soil

## Abstract

Nitrogen-based fertilizers are crucial in agriculture for maintaining soil health and increasing crop yields. Soil microorganisms transform nitrogen from fertilizers into ${NO}_{3}^{-}$–N, which is absorbed by crops. However, some nitrogen is converted to nitrous oxide (N_2_O), a greenhouse gas with a warming potential about 300-times greater than carbon dioxide (CO_2_). Agricultural activities are the main source of N_2_O emissions. Monitoring N_2_O can enhance soil health and optimize nitrogen fertilizer use, thereby supporting precision agriculture. To achieve this, we developed ionic liquid-gated graphene field-effect transistor (FET) sensors to measure N_2_O concentrations in agricultural soil. We first fabricated and tested the electrical characteristics of the sensors. Then, we analyzed their transfer characteristics in our developed N_2_O evaluation system using different concentrations of N_2_O and air. The sensors demonstrated a negative shift in transfer characteristic curves when exposed to N_2_O, with a Dirac point voltage difference of 0.02 V between 1 and 10 ppm N_2_O diluted with pure air. These results demonstrate that the ionic liquid-gated graphene FET sensor is a promising device for N_2_O detection for agricultural soil applications.

## 1. Introduction

Nitrogen is the most critical limiting factor for healthy and stable crop growth [1,2]. However, the nitrogen transformation cycle propagated by microorganisms causes the formation of multiple unwanted chemical forms for plant growth, leading to imbalanced fertilization. While a lack of nitrogen fertilizer limits crop growth, excessive fertilizer causes ${NO}_{3}^{-}$–N leaching into groundwater and nitrous oxide (N_2_O) air pollution [3]. Although carbon dioxide (CO_2_) is a prominent greenhouse gas, N_2_O is a significant contributor to climate change, as it is an ozone-depleting substance. One pound of N_2_O is approximately 300-times more harmful to global warming than one pound of CO_2_ [4,5,6,7]. Moreover, a rapid increase in the global emissions of N_2_O to 40% is reported to have occurred from 1980 to 2020 [6].

The primary anthropogenic source of N_2_O is agricultural soil management activities. Soil microorganisms generate N_2_O through the processes of nitrification and denitrification. With rapid population growth, agricultural activity is on the rise. By 2050, the production and consumption of major field crops are projected to increase by 75% [8]. Research on monitoring N_2_O levels in agricultural soil has been widely conducted to optimize soil health and manage fertilization amounts effectively. The most common measurement method involves installing a gas collection chamber in the soil and analyzing the collected gas using gas chromatography [7,9]. However, gas chromatography has disadvantages, including its large size and high cost, which make it impractical for real-time local monitoring.

Graphene field-effect transistor (FET) gas sensors based on microelectromechanical systems (MEMSs) show great promise due to their compact size, high mobility, and outstanding sensitivity [10,11,12,13,14,15,16]. The use of back-gated and front-gated structures enhances their versatility for biosensing applications [17]. Currently, graphene FET devices come in various shapes and sizes, allowing for the detection of a wide range of target gases. However, the prolonged recovery time for the desorption of adsorbed substances limits their effectiveness for real-time monitoring. In contrast, ionic liquid-gated graphene FETs offer benefits such as shorter release times and lower gate voltages, making them a promising option for advancing gas-sensing technology.

Nitrogen cycle monitoring in agricultural soil focuses on two targets: dissolved ${NO}_{3}^{-}$–N in soil water and N_2_O gas released from the soil. To detect dissolved ${NO}_{3}^{-}$–N, a larger amount of ionic liquid (200 nL to 600 nL) is preferred in order to minimize water interference. Ionic liquid-gated graphene FET sensors with a hydrophobic CYTOP layer can accommodate these amounts, but this process may damage the graphene channels [13]. In contrast, N_2_O is emitted as a gas. Sensors using less than 100 nL of ionic liquid effectively detect N_2_O.

In this study, we developed a new high-sensitivity sensor that solves the problems of the sensor in the previous paper. Furthermore, we developed two types of ionic liquid-gated graphene FET sensors and evaluated how processing affects their electrical properties. We also created a new evaluation system to investigate the response characteristics of N_2_O gas, which is a greenhouse gas, specifically examining the shift in transfer characteristic curves when exposed to N_2_O concentrations of 0.02 ppm, 1 ppm, and 10 ppm.

## 2. Principle of N_2_O Detection Using Ionic Liquid-Gated Graphene FET

**Figure 1:**
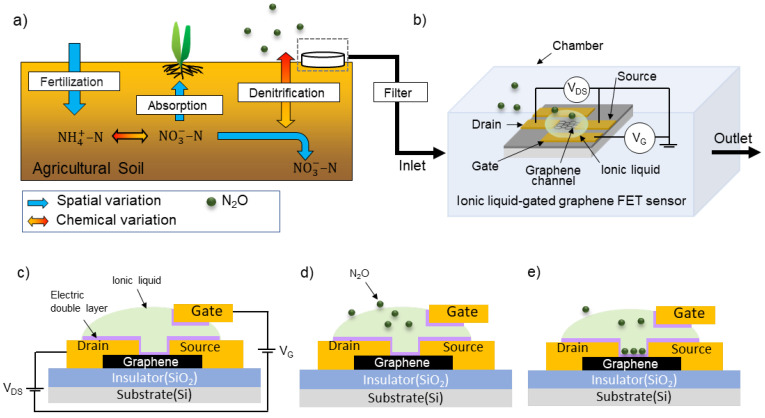
Schematic of N_2_O monitoring in agricultural soil using an ionic liquid-gated graphene FET sensor: (**a**) spatial variation and chemical variation in nitrogen in agricultural soil; (**b**) schematic illustration and (**c**–**e**) working principle of an ionic liquid-gated graphene FET sensor in chamber.

Figure 1 illustrates a schematic of N_2_O monitoring in agricultural soil using an ionic liquid-gated graphene FET sensor. Figure 1a explains nitrogen’s spatial and chemical variations in agricultural soil. Nitrogen-based fertilizers are commonly used to maintain healthy soil and achieve higher crop yields. Nitrogen undergoes complex transformations through biochemical reactions driven by microorganisms in the soil. These soil microorganisms convert the nitrogen in fertilizers into ${NO}_{3}^{-}$–N, which is then absorbed by crops. However, some of this nitrogen is reduced to N_2_O and escapes into the atmosphere.

N_2_O concentrations are currently detected using a static chamber technique, which involves gas chromatography with an electron capture detector or quantum cascade laser spectrometry combined with isotope ratio mass spectrometry. This technique involves placing a gas collection chamber on agricultural soil to capture gases. The gas is transferred through a PTFE tube and filtered to remove dust and moisture CO_2_ before entering the N_2_O evaluation chamber. Akiyama et al. provided detailed descriptions of this evaluation system in several studies [18,19]. Our study uses the same static chamber technique but adds an ionic liquid-gated graphene FET to evaluate N_2_O concentrations.

Figure 1b illustrates a schematic diagram of a sensor chamber constructed from an airtight container. The design of the ionic liquid-gated graphene FET includes a graphene channel; electrodes for the source, drain, and gate; and an ionic liquid droplet covering both the electrodes and the graphene channels. Graphene is an exceptional material because of its unique properties and structure. Its application in gas sensors has been widely studied, particularly for its sensitivity at the parts-per-billion (ppb) detection level [11].

Ionic liquids are promising gate-source materials for graphene FETs due to their ability to enhance gas absorption at low voltages and concentrations. Ionic liquids made from alkyl imidazolium cations and tetrafluoroborate anions are efficient for gas sensing, effectively absorbing and converting gases, such as NO*_x_*, CO_2_, C_2_H_6_, and N_2_. This effectiveness is attributed to their low vapor pressure, structural flexibility, and excellent recyclability [20,21]. Pereira et al. (2014) found that Henry’s law constant for N_2_O solubility in [C_4_mim][BF_4_] ionic liquid is 8.60 MPa at room temperature, compared to 7.60 MPa for CO_2_ [22].

Ionic liquids offer significant stability, making them ideal for gas-sensing applications. Even small volumes (in the range of nanoliters) can achieve a large surface-to-volume ratio, ensuring excellent mechanical strength, even when the sensor is tilted or turned upside down. Furthermore, ionic liquids are largely unaffected by most non-polar solvents [23]. Their non-volatility, high thermal stability, and electrochemical stability make them durable enough for use in harsh conditions over extended operational periods [24].

Figure 1c–e illustrate the working principle of an ionic liquid-gated graphene FET. When the gate voltage is applied to the ionic liquid, a non-conductive electric double layer is formed near the graphene channel surface, resulting in a field-effect transistor, with the ionic liquid as the gate (Figure 1c). When the positive gate voltage is applied to the ionic liquid, cations in the ionic liquid and electrons in the graphene attract each other. In contrast, when the negative voltage is applied to the ionic liquid, the anions in the ionic liquid interact with the holes in the graphene.

Then, the sensor is exposed to N_2_O gas; the N_2_O molecules absorbed onto the ionic liquid’s surface are shown in Figure 1d. The N_2_O molecules diffused inside the ionic liquid are doped into the graphene channel as charges (Figure 1e). This process leads to changes in the electrical properties of the graphene FET, as the electrical conductivity of the graphene channel increases with the amount of hole or electron charge [25]. Thus, the N_2_O concentration can be detected by measuring the shift in the transfer characteristic curve.

## 3. Development of Ionic Liquid-Gated Graphene FET Sensors

### 3.1. Fabrication

**Figure 2:**
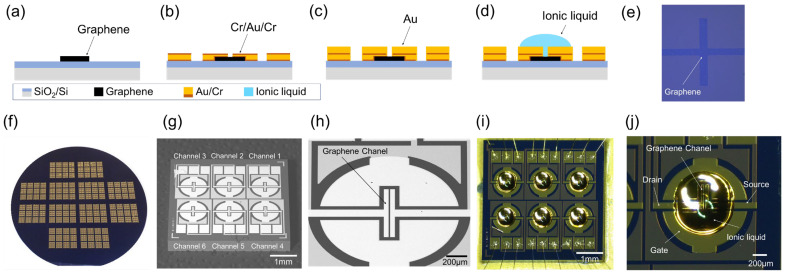
Fabrication process of an ionic liquid-gated graphene FET sensor: (**a**) graphene etching using O_2_ plasma; (**b**) Cr/Au/Cr deposition using vapor deposition and patterning using a lift-off process; (**c**) 100 nm Au coating on electrodes using a lift-off process; (**d**) ionic liquid dropped; (**e**) photograph of graphene pattern formed using O_2_ plasma; (**f**) photograph of a processed wafer; (**g**) SEM image of a fabricated sensor chip containing 6 graphene FETs with the same graphene channel; (**h**) enlarged SEM image of channel 1; (**i**) photograph of an ionic liquid-gated graphene FET sensor; (**j**) enlarged photograph of channel 1.

Two types of ionic liquid-gated graphene FETs, one with a hydrophobic layer and one without, were fabricated in this study. We first discuss the fabrication process for the ionic liquid-gated graphene FET without the hydrophobic layer. Figure 2 illustrates the device fabrication process flow, as well as presenting photographs and scanning electron microscope (SEM) images of the completed ionic liquid-gated graphene FET sensor without the hydrophobic layer. Fabrication began by etching graphene patterns from 4-inch, single-layer CVD-grown graphene on a SiO_2_/Si substrate using O_2_ plasma (Figure 2a). Next, source, drain, and gate electrodes were deposited and patterned using a chromium/gold/chromium (Cr/Au/Cr) configuration, with thicknesses of 3 nm, 60 nm, and 5 nm, respectively (Figure 2b). To prevent contact between the chromium and ionic liquid, a 100 nm thick layer of gold was deposited on the electrodes and bonding pads (Figure 2c). All deposition and patterning processes were carried out using vapor deposition and a lift-off technique. Finally, 100 nL of the ionic liquid of 1-propyl-3-methylimidazolium tetrafluoroborate ([PMIM][BF_4_]) was dispensed onto the graphene channel using an auto-dispenser (IM 300 Microinjector, NARISHIGE Co., Ltd., Tokyo, Japan) (Figure 2d).

An enlarged photograph of the graphene pattern formed by the O_2_ plasma is presented in Figure 2e. Figure 2f presents the final grid layout of 5 mm × 5 mm sensor chips on a 4-inch SiO_2_/Si wafer. Each sensor chip contains six graphene FETs. We designed three different sizes of graphene channels: 10 μm × 50 μm, 10 μm × 500 μm, and 20 μm × 500 μm. An SEM image of a sensor chip with graphene channels of the same size (20 μm × 500 μm), labeled as channels 1 to 6, is shown in Figure 2g. An enlarged SEM image of channel 1 of the graphene FET is displayed in Figure 2h. Finally, Figure 2i,j show photographs of the final fabricated ionic liquid-gated graphene FET and an enlarged view of channel 1 with 100 nL of ionic liquid, respectively.

**Figure 3:**
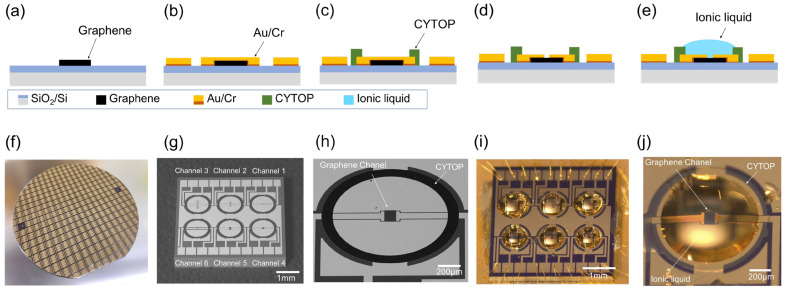
Fabrication process of an ionic liquid-gated graphene FET sensor with a hydrophobic layer: (**a**) graphene etching using O_2_ plasma; (**b**) Au/Cr deposition using vapor deposition and patterning using a lift-off process; (**c**) CYTOP formation; (**d**) etching of Au/Cr on graphene channel; (**e**) ionic liquid dropped; (**f**) photograph of a processed wafer with a hydrophobic layer; (**g**) SEM image of a fabricated sensor chip containing 6 FETs with different graphene channel sizes; (**h**) enlarged SEM image of channel 5; (**i**) photograph of an ionic liquid-gated graphene FET sensor; (**j**) enlarged photograph of channel 5.

In Figure 3a–e, we describe the fabrication process of an ionic liquid-gated graphene FET with CYTOP as a hydrophobic layer. The purpose of the hydrophobic layer is to incorporate more ionic liquid into the graphene FET. The process began by etching graphene patterns using O_2_ plasma from 4-inch, single-layer CVD-grown graphene on a SiO_2_/Si substrate, as shown in Figure 3a. After that, source, drain, and gate electrodes were deposited and patterned through vapor deposition and a lift-off technique, using a gold/chromium (Au/Cr) configuration with thicknesses of 36 nm and 4 nm, respectively (Figure 3b). Subsequently, a 1 mm diameter hollow-shaped hydrophobic layer, made from CYTOP^®^ (CTL-809M, Asahi Glass Co., Ltd., Tokyo, Japan), was deposited around the graphene channel, as illustrated in Figure 3c. Next, the Au/Cr layer on the graphene channel was removed using a wet etching process (Figure 3d). Finally, 200 nL of the ionic liquid [PMIM][BF4] was added to the graphene channel using an auto-dispenser (Figure 3e). More details of the fabrication process can be found in previously published work [13].

Figure 3f shows a photograph of a grid containing 100 pieces of 5 mm × 5 mm graphene FET sensor chips on the 4-inch wafer. Figure 3g,h provide SEM images of the fabricated ionic liquid-gated graphene FET sensor with the hydrophobic layer. Each sensor chip included six graphene FETs with various graphene channel dimensions: 20 μm × 50 μm, 20 μm × 250 μm, 20 μm × 500 μm, 100 μm × 50 μm, and 100 μm × 100 μm. Figure 3i,j present photographs of the final fabricated ionic liquid-gated graphene FET and an enlarged image of channel 5 containing 200 nL of ionic liquid.

### 3.2. Electrical Characteristics

**Figure 4:**
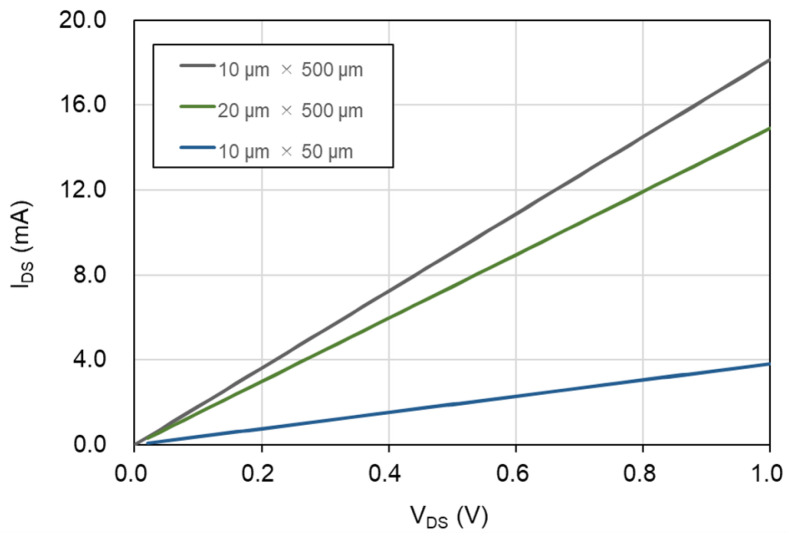
Current–voltage characteristics of the source–drain electrodes of the ionic-gated graphene FET sensor without a hydrophobic layer. The Y-axis of I_DS_ represents the source–drain current. The X-axis of V_DS_ represents the source–drain voltage.

We examined the current–voltage (I-V) characteristics of the source–drain electrodes in the ionic liquid-gated graphene FETs, both with and without a hydrophobic layer, using a source meter (Keithley 2450, Tektronix, Inc., Tokyo, Japan) at room temperature. Figure 4 displays the I-V characteristics of the ionic liquid-gated graphene FETs, comparing the conditions without the hydrophobic layer. A linear relationship was observed between the source–drain current (I_DS_) and the source–drain voltage (V_DS_) from 0 V to 1 V. The measured conductance depended on the dimensions of the graphene channels; specifically, a wider and shorter graphene channel exhibited higher conductance.

The sheet resistance measurements of the ionic liquid-gated graphene FETs without a hydrophobic layer were 1312 Ω/sq for a channel size of 10 μm × 50 μm, 1382 Ω/sq for 10 μm × 500 μm, and 1676 Ω/sq for 20 μm × 500 μm. In contrast, the sheet resistance measurements of the ionic liquid-gated graphene FETs with a hydrophobic layer ranged from 4580 to 8391 Ω/sq. The CYTOP well was used to contain more electrolyte; this was a basic practical choice. Although the hydrophobic layer allows for the incorporation of more ionic liquid into the graphene FET, the process of forming this layer damages the graphene channels, resulting in a higher sheet resistance. In summary, the lower sheet resistance observed in the ionic liquid-gated graphene FETs without a hydrophobic layer indicates the presence of higher-quality graphene channels.

## 4. Evaluation of N_2_O Detection Using the Ionic Liquid-Gated Graphene FET Sensors

### 4.1. Measurements

**Figure 5:**
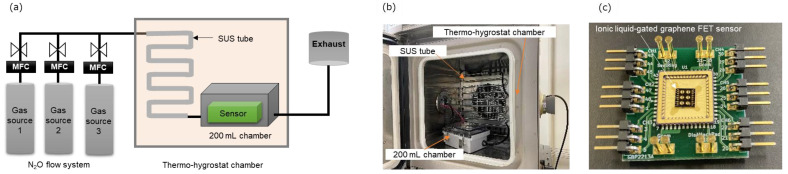
(**a**) Schematic view of our N_2_O evaluation system; (**b**) photograph of the thermos-hydrostat chamber; (**c**) evaluation module of the wire-bonded sensor on a ceramic package.

We developed an evaluation system for detecting N_2_O using ionic liquid-gated graphene FETs. Figure 5a shows a schematic of the N_2_O evaluation system, which consists of two main components: the N_2_O flow system and a thermo-hygrostat chamber. In the N_2_O flow system, three gas sources are connected to the mass flow controller (MFC), which is regulated by a two-way valve in the gas lines leading to the thermo-hygrostat chamber. The ionic liquid-gated graphene FET is set up in a 200 mL chamber, connected to a stainless-steel tube (SUS) for the gas inlet, and it has an exhaust for the gas outlet. An actual photograph of the thermo-hygrostat chamber is provided in Figure 5b. Figure 5c illustrates the evaluation module for the ionic liquid-gated graphene FET sensor. The evaluation of N_2_O was conducted at a specific flow rate entering the 200 mL chamber. A V_DS_ was applied, and the I_DS_ was recorded using a source meter (Keithley 2410, Tektronix, Inc., Tokyo, Japan). Meanwhile, the gate voltage was scanned with a source measure unit (GS610, Yokogawa Electric Corporation, Tokyo, Japan).

The current concentration of N_2_O in the atmosphere is 0.1 ppm. The amount of N_2_O emitted from the soil depends on the quantity of fertilizer used, which varies for different crops and is influenced by the region’s soil composition. However, this emission can reach a maximum concentration of approximately 10 ppm [26].

Table 1 presents the N_2_O measurement conditions for the ionic liquid-gated graphene FET sensors. Measurement 1 was conducted on channel 2 of the 200 nL [PMIM][BF_4_]-ionic liquid-gated graphene FET sensor with a hydrophobic layer at 25 ± 1 °C. We first examined the N_2_O response by introducing 10 ppm N_2_O diluted with N_2_ from gas source 1. The transfer characteristic curves of the sensor were recorded in the following sequence: starting with air, followed by 10 ppm N_2_O diluted with N_2_, and again with air, all at a flow rate of 1000 sccm for 10 min in each cycle. The air passed through a filter (MFU50-66, NIHON PISCO Co., Ltd., Okaya, Nagano, Japan) before entering the 200 mL chamber, facilitated by a pump and an MFC. The I_DS_ was recorded every 2 s over a gate voltage range of −1 V to 1 V at a scan rate of 0.1 V and a V_DS_ of 10 mV.

Measurement 2 was conducted on channel 1 of the 100 nL [PMIM][BF_4_]-ionic liquid-gated graphene FET sensor without a hydrophobic layer at 25 ± 1 °C. The graphene channel size was 10 μm × 50 μm. We examined the N_2_O response by introducing 1 ppm N_2_O diluted with pure air from gas source 2 and 10 ppm N_2_O diluted with pure air from gas source 1. The sensor’s transfer characteristic curves were recorded in the following sequence: starting with air, followed by 1 ppm N_2_O diluted with pure air, again with air, and finally with 10 ppm N_2_O diluted with pure air. All measurements were conducted at a flow rate of 1000 sccm for 30 min in each cycle. The I_DS_ was recorded every 2 s over a gate voltage range of −0.1 V to 0.7 V at a scan rate of 0.1 V and a V_DS_ of 10 mV. The measurements were taken at 5, 10, and 30 min in the flowing gas for each gas.

Measurement 3 was conducted on channel 2 of the 100 nL [PMIM][BF_4_]-ionic liquid-gated graphene FET sensor without a hydrophobic layer at 25 ± 1 °C. The graphene channel size was 10 μm × 50 μm. We examined the N_2_O response by introducing 0.02 ppm N_2_O diluted with pure air from gas source 3, 1 ppm N_2_O diluted with pure air from gas source 2, and 10 ppm N_2_O diluted with pure air from gas source 1. The sensor’s transfer characteristic curves were recorded in the following sequence: starting with air, followed by 0.02 ppm N_2_O diluted with pure air, 1 ppm N_2_O diluted with pure air, and finally with 10 ppm N_2_O diluted with pure air. All measurements were conducted at a flow rate of 1000 sccm for 5 min in each cycle. The I_DS_ was recorded every 2 s over a gate voltage range of 0.1 V to 0.6 V at a scan rate of 0.1 V and a V_DS_ of 10 mV. The measurements were taken at 5 min in the flowing gas for each gas.

To investigate the selectivity of this sensor, its responses to CO_2_ and H_2_O were measured. We chose CO_2_ and H_2_O because they are the most competitive gases in practical soil fields that change with soil conditions. Measurement 4 was conducted on channel 1 of the 100 nL [PMIM][BF_4_]-ionic liquid-gated graphene FET sensor without a hydrophobic layer at 25 ± 1 °C. The graphene channel size was 10 μm × 50 μm. We examined the N_2_O and CO_2_ responses by introducing 1 ppm N_2_O diluted with pure air from gas source 2, 10 ppm N_2_O diluted with pure air from gas source 1, and 50,000 ppm CO_2_ diluted with pure air from gas source 3. The sensor’s transfer characteristic curves were recorded in the following sequence: starting with air, followed by 0.02 ppm N_2_O diluted with pure air, 1 ppm N_2_O diluted with pure air, and finally with 50,000 ppm CO_2_ diluted with pure air. All measurements were conducted at a flow rate of 1000 sccm for 5 min in each cycle. The I_DS_ was recorded every 2 s over a gate voltage range of −1.0 V to 0.5 V at a scan rate of 0.1 V and a V_DS_ of 10 mV. The measurements were taken at 5 min in the flowing gas for each gas.

### 4.2. Results

**Figure 6:**
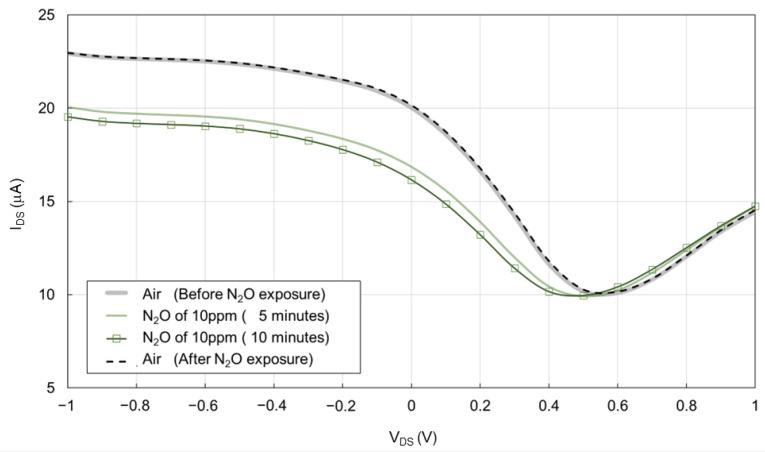
Transfer characteristic curve of the 200 nL [PMIM][BF_4_]-ionic liquid-gated graphene FET sensor with a hydrophobic layer exposed to air, followed by 10 ppm N_2_O diluted with N_2_ and then by air at a constant 1000 sccm for 10 min. The Y-axis of I_DS_ represents the source–drain current. The X-axis of V_DS_ represents the source–drain voltage.

Figure 6 shows the transfer characteristic curve of the 200 nL [PMIM][BF_4_]-ionic liquid-gated graphene FET sensor with a hydrophobic layer during measurement 1. A typical ambipolar transfer characteristic curve, a primary feature of graphene FETs, was observed [14,15]. At a 0.5 V Dirac point in air, the I_DS_ increased in both positive and negative directions. The transfer characteristic curve shifted significantly in the negative direction during N_2_O exposure. When N_2_O was no longer present, the transfer characteristic curve shifted back to the baseline condition after 10 min without any heat treatment.

**Figure 7:**
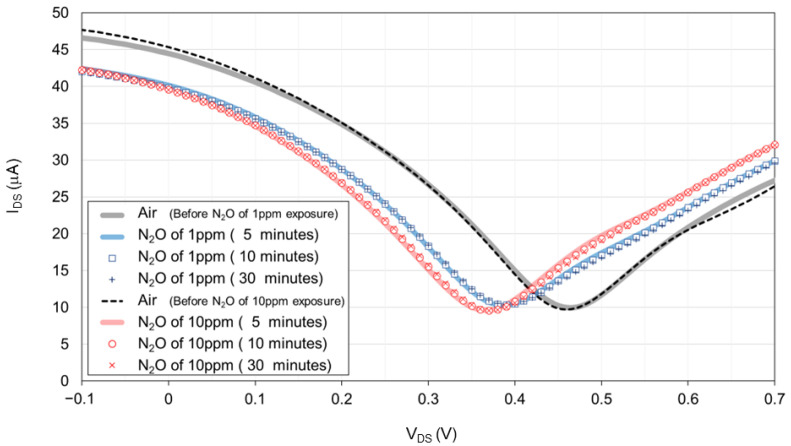
Transfer characteristic curve of the 100 nL [PMIM][BF_4_]-ionic liquid-gated graphene FET sensor without a hydrophobic layer exposed to air, 1 ppm N_2_O diluted with pure air, air, and lastly 10 ppm N_2_O diluted with pure air at 1000 sccm for 30 min. The Y-axis of I_DS_ represents the source–drain current. The X-axis of V_DS_ represents the source–drain voltage.

Figure 7 presents the transfer characteristic curve of the 100 nL [PMIM][BF_4_]-ionic liquid-gated graphene FET sensor without a hydrophobic layer during measurement 2. The transfer characteristic curves for 1 ppm and 10 ppm N_2_O remained consistent across the measurements at 5, 10, and 30 min. Upon exposure to N_2_O in the chamber, a negative shift was observed, moving from the 0.47 V Dirac point in air to 0.39 V with 1 ppm N_2_O and 0.37 V with 10 ppm N_2_O. This resulted in a 0.02 V difference between the Dirac points for the 1 ppm and 10 ppm N_2_O exposure. The evaluated response time was within 5 min, indicating that the ionic liquid-gated graphene FET sensor is highly sensitive to low concentrations of N_2_O gas.

**Figure 8:**
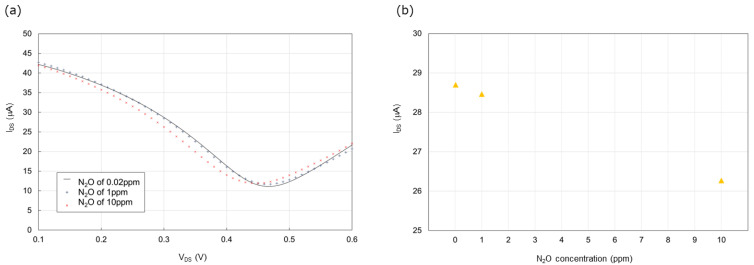
(**a**) Transfer characteristic curve of the 100 nL [PMIM][BF_4_]-ionic liquid-gated graphene FET sensor without a hydrophobic layer exposed to 0.02 ppm N_2_O diluted with pure air and 1 ppm and 10 ppm N_2_O diluted with pure air at 1000 sccm for 5 min. The Y-axis of I_DS_ represents the source–drain current. The X-axis of V_DS_ represents the source–drain voltage; (**b**) N_2_O concentration dependence of source–drain current at a gate voltage of 0.3 V. The I_DS_ at a gate voltage of 0.3 V of 0.02 ppm N_2_O diluted with pure air and 1 ppm and 10 ppm N_2_O diluted with pure air in Figure 8a were plotted.

Figure 8a presents the transfer characteristic curve of the 100 nL [PMIM][BF_4_]-ionic liquid-gated graphene FET sensor without a hydrophobic layer during measurement 3. The effect of the N_2_O concentration on the I_DS_ at a gate voltage of 0.3 V is shown in Figure 8b. The I_DS_ decreased as the N_2_O concentration increased. The I_DS_ was 28.70 μA at 0.02 ppm, 28.46 μA at 1 ppm, and 26.27 μA at 10 ppm N_2_O.

**Figure 9:**
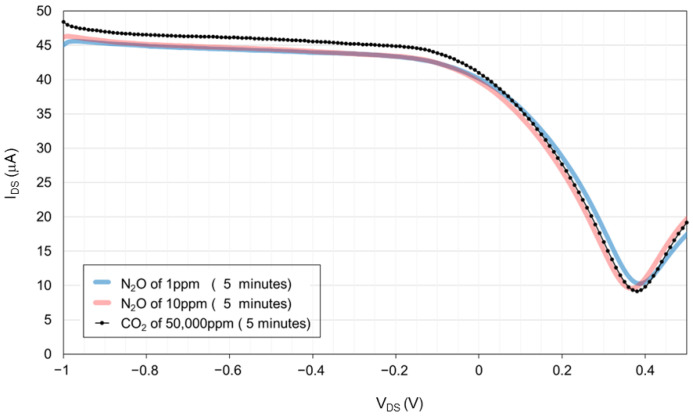
Transfer characteristic curve of the 100 nL [PMIM][BF_4_]-ionic liquid-gated graphene FET sensor without a hydrophobic layer exposed to 1 ppm and 10 ppm N_2_O diluted with pure air and 50,000 ppm CO_2_ diluted with pure air at 1000 sccm for 5 min. The Y-axis of I_DS_ represents the source–drain current. The X-axis of V_DS_ represents the source–drain voltage.

Figure 9 presents the transfer characteristic curve of the 100 nL [PMIM][BF_4_]-ionic liquid-gated graphene FET sensor without a hydrophobic layer during measurement 4. The I_DS_ of 1ppm N_2_O and 1ppm N_2_O at a gate voltage of −0.3 V was 43.78 μA and 43.76 μA, respectively. In contrast, the I_DS_ of 50,000 ppm CO_2_ at a gate voltage of −0.3 V was 45.19 μA.

### 4.3. Discussion

Research shows that N_2_O sensors for agricultural soil must measure between 0.3 ppm and 10 ppm [26]. Our findings reveal that the [PMIM][BF_4_]-ionic liquid-gated graphene FET sensor is very sensitive to N_2_O, as demonstrated by the significant negative shift in the lower gate voltage observed from 0.02 ppm, 1 ppm, and 10 ppm N_2_O diluted with pure air. We measured a detection sensitivity for N_2_O of 0.246 μA/ppm in the I_DS_ between 0.02 ppm and 10 ppm N_2_O diluted with pure air at a gate voltage of 0.3 V.

When N_2_O was absent, the sensor’s transfer characteristic curve returned to its baseline condition without heat treatment, as shown in Figure 6 and Figure 7. Additionally, the gate voltage for the [PMIM][BF_4_]-ionic liquid-gated graphene FET sensors was much lower than that for the graphene FET sensors. This is promising for reducing power consumption in sensor networks that monitor nitrogen cycles in agricultural soil.

Both 1 ppm and 10 ppm N_2_O showed consistent transfer characteristic curves at measurements taken at 5 and 10 min, which means that the sensor completely adsorbs N_2_O within 5 min. The negative shift in the Dirac point for different N_2_O concentrations highlights the sensor’s high sensitivity. The quick response time and ability to detect low levels of N_2_O (in the ppm range) prove that the 100 nL [PMIM][BF4]-ionic liquid-gated graphene FET sensor is effective for monitoring N_2_O in agricultural soil.

Moreover, the transfer characteristic curve of the ionic liquid-gated graphene FET sensor showed positive and negative shifts after H_2_O exposure and release [14]. For our 100 nL [PMIM][BF_4_]-gated graphene FET sensor, the consistent and shift-back behavior of the transfer characteristic curves indicates that the sensor’s humidity effect was eliminated in 5 min. We believe that ionic liquid-gated graphene FET sensors have strong potential for use in agriculture. They are compact, low-cost, and energy-efficient while remaining highly sensitive. Although more research is needed, these sensors will likely meet regulatory needs for monitoring nitrogen cycles in agricultural soil.

For practical use of the sensor, selectivity to ${NO}_{3}^{-}$–N, ${NH}_{4}^{+}$–N, and CO_2_ is required. In the case of CO_2_, the difference in the I_DS_ between 1ppm N_2_O and 50,000 ppm CO_2_ at a gate voltage of −0.3 V is 1.43 μA. In contrast, the difference in the I_DS_ between 1ppm N_2_O and 10 ppm N_2_O at a gate voltage of −0.3 V is 0.02 μA, as shown in Figure 9. CO_2_ absorption affects the thickness of the non-conductive electric double layer because of the change in ionic strength [12]. The selectivity between N_2_O and CO_2_ seems to be obtained by the change in the I_ds_ when a negative voltage is applied to the gate electrode.

**Figure 10:**
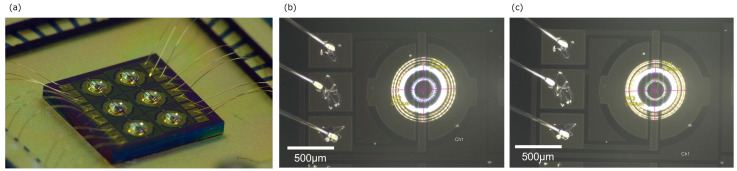
(**a**) Photograph of parylene C-coated ionic-gated graphene FET sensor; (**b**) enlarged micrograph of ch1 of parylene C-coated ionic-gated graphene FET sensor; (**c**) enlarged micrograph of ch1 before the application of the parylene C coating.

For practical applications, the selectivity of our sensor to ${NO}_{3}^{-}$–N and ${NH}_{4}^{+}$–N is also essential. As ${NO}_{3}^{-}$–N and ${NH}_{4}^{+}$–N dissolve in soil solution, the sensor must have a filter function to remove the soil solution. Currently, the selective detection of N_2_O is achieved by removing the soil solution using a small external Perma Pure filter (GL Sciences Inc., Tokyo, Japan) [27,28]. Although this filter can be applied as a filter for our sensor, we developed a sensor with a thin parylene C nanofilm on the ionic liquid and investigated the selective permeation property of water to make the sensor small. Figure 10a shows the parylene C-coated ionic-gated graphene FET sensor. A parylene C monomer of 0.5 g was heated to 135 °C, and a film was prepared on ionic liquid using chemical vapor deposition (CVD). The thickness of the parylene film was 350 nm, measured using an interferometer. An enlarged micrograph of the parylene C-coated ionic-gated graphene FET sensor and a micrograph of the sensor before the application of the parylene C coating are shown in Figure 10b,c, respectively. A thin parylene C nanofilm was coated on the ionic liquid without changes in the ionic liquid shape.

**Figure 11:**
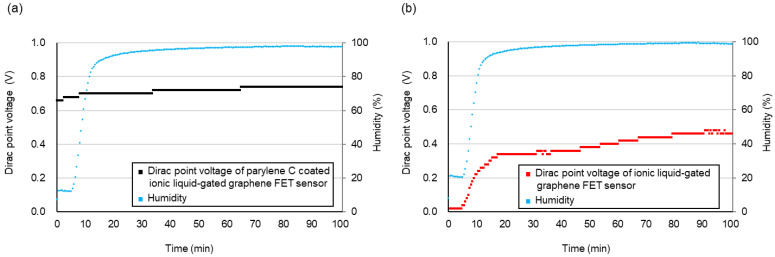
Dirac point voltage shift of 100 nL [PMIM][BF4]-ionic liquid-gated graphene FET sensor exposed to pure water vapor: (**a**) with parylene C coating; (**b**) without parylene C.

The selective permeation property of the pure water of the parylene C-coated ionic-gated graphene FET is shown in Figure 11a. Figure 11b shows the Dirac point voltage shift of the ionic liquid-gated graphene FET without parylene C to compare the conditions with and without parylene C. Measurements were conducted on channel 1 of the 100 nL [PMIM][BF_4_]-ionic liquid-gated graphene FET sensor at 23 ± 1 °C. The graphene channel size was 10 μm × 50 μm. We examined the pure water response by introducing pure water vapor after evacuating the chamber for 20 min. The transfer characteristic curves were recorded every 30 s over a gate voltage range of −1 V to 1 V at a scan rate of 0.1 V and a V_DS_ of 10 mV, and Dirac point voltages were plotted. Upon exposure to pure water vapor in the chamber, a positive shift in the Dirac point was observed, with a 0.52 V shift for a change in humidity from 8.2% to 98.9% in the [PMIM][BF_4_]-ionic liquid-gated graphene FET sensor without parylene C. In contrast, the Dirac point shifted by 0.08 V for the parylene C-coated [PMIM][BF_4_]-ionic liquid-gated graphene FET sensor as the humidity changed from 7.4% to 98.0%. Parylene C had a low permeability coefficient for H_2_O vapor and showed excellent barrier properties that prevent the penetration of water. In our research, investigations of the selectivity of our sensor to various gases are currently ongoing by using different ionic liquids, as well as by introducing a thin nanofilm on the ionic liquid as the filter. The results and discussion will be published in our future publications.

## 5. Conclusions

This study discussed the basic characteristics of ionic liquid-gated graphene FETs for sensing N_2_O gas in agricultural soil. We fabricated sensors with 100 nL of [PMIM][BF_4_]-ionic liquid-gated graphene FETs, which exhibited a sheet resistance of 1300 Ω/sq. To assess the sensor’s response to N_2_O, we established a system that included a flow controller for N_2_O and a thermo-hygrostat chamber. We observed that the transfer characteristic curves of the [PMIM][BF_4_]-ionic liquid-gated graphene FET sensor shifted upon exposure to N_2_O. Notably, the difference in the Dirac point of 0.02 V between 1 ppm and 10 ppm N_2_O diluted in pure air indicates that the ionic liquid-gated graphene FET sensor is a promising device for N_2_O detection in agricultural soils. Further fundamental research and dedicated experiments are essential to fully understand the sensor’s sensitivity and selectivity. Ongoing evaluations aim to explore the performance of ionic liquid-gated graphene FET sensors in various practical applications.

## Figures and Tables

**Table 1:** List of measurement conditions of N_2_O evaluation of the ionic liquid-gated graphene FET sensors.

Measurement	Graphene FET	Gas Source 1	Gas Source 2	Gas Source 3	Flow Rate
1	200 nL of [PMIM][BF_4_] with a hydrophobic layer	N_2_O of 10 ppm diluted with N_2_	-	-	1000 sccm
2	100 nL of [PMIM][BF_4_] Channel 1	N_2_O of 10 ppm diluted with pure air	N_2_O of 1 ppm diluted with pure air	-	1000 sccm
3	100 nL of [PMIM][BF_4_] Channel 2	N_2_O of 10 ppm diluted with pure air	N_2_O of 1 ppm diluted with pure air	N_2_O of 0.02 ppm diluted with pure air	1000 sccm
4	100 nL of [PMIM][BF_4_] Channel 1	N_2_O of 10 ppm diluted with pure air	N_2_O of 1 ppm diluted with pure air	CO_2_ of 50,000 ppm diluted with pure air	1000 sccm

## Data Availability

Data are contained within the article.
